# Ectopic “Ectopic” Gastric Mucosa

**DOI:** 10.3390/diagnostics14111162

**Published:** 2024-05-31

**Authors:** Adeel Haq, Amin Haghighat Jahromi

**Affiliations:** Mallinckrodt Institute of Radiology, Washington University in St. Louis, St. Louis, MO 63110, USA; aminj@wustl.edu

**Keywords:** Meckel’s diverticulum, ectopic gastric mucosa, Tc-99m pertechnetate, omphalo-mesenteric duct remnant

## Abstract

Meckel’s diverticulum is a developmental GI anomaly. It is a remnant of the omphalomesenteric duct (vitelline duct) and the most common congenital anomaly found in the small intestine. It contains ectopic/heterotopic gastric mucosa in half of the cases. Imaging investigations for diagnosing Meckel’s diverticulum may include a plain radiography; however, this has a very limited diagnostic value. A blind-ending fluid-filled structure can sometimes be seen with sonography, but again, this technique’s diagnostic value is limited due to multiple factors. A CT scan may be helpful in localizing the bleeding diverticulum, which can be better visualized with CT enterography. Diverticula containing gastric mucosa can be diagnosed with a higher sensitivity with Tc-99 scintigraphy. The typical location of Meckel’s diverticulum is within two feet of the ileocecal valve; thus, ectopic gastric mucosal uptake is typically seen in the lower right quadrant in scintigraphy. We present a rare case of Tc-99 pertechnetate scintigraphy showing ectopic gastric mucosa in the upper mid abdomen, which was surgically proven to be at the mid ileum. To our knowledge, there is no ectopic Meckel’s diverticulum case published in the literature. Familiarity with this atypical imaging presentation of relatively common ectopic gastric mucosa may help the radiologists in the timely diagnosis and management of the patient.

**Figure 1:**
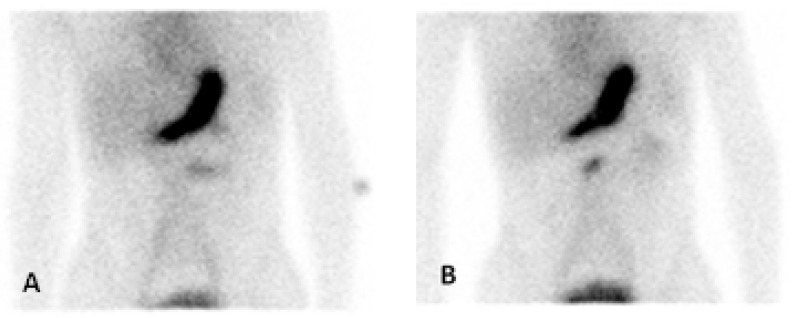
Early anterior (**A**) and delayed anterior (**B**) planar images with Tc-99m pertechnetate showing abnormal focus of activity in the superior mid abdomen with peristalsis-like movement on dynamic cine images suggesting ectopic gastric mucosa in Meckel’s diverticulum. A 16-year-old boy presented with several days of bloody stools and anemia, indicative of gastrointestinal bleeding. He had painless bloody stools. His hemoglobin was 10.3 mg/dL at the first presentation, which dropped to 5.9 mg/dL and so he received packed red blood cells. He did not have pain, diarrhea, or vomiting. CT (Computed Tomography) of the abdomen and pelvis with contrast was obtained which did not show definite vascular or gastrointestinal abnormality. Nuclear medicine Meckel’s scan was obtained with Tc-99m pertechnetate which showed an abnormal focus of tracer activity in the mid abdomen, suggesting “ectopic” Meckel’s diverticulum. The patient underwent laparoscopic diverticulectomy the next day and a diverticulum was found at the anti-mesenteric border of the mid ileum. The surgical pathology of the specimen showed a true diverticulum with the ectopic oxyntic type of gastric mucosa, which is consistent with Meckel’s diverticulum. Meckel’s diverticulum is a developmental GI anomaly [1]. It is a remnant of the omphalomesenteric duct (vitelline duct) [2] and the most common congenital anomaly in the small intestine [3]. It contains ectopic/heterotopic gastric mucosa in approximately half of the cases [4]. The typical location of Meckel’s diverticulum is within two feet of the ileocecal valve [5]; thus, ectopic gastric mucosal uptake is typically seen in the lower right quadrant in scintigraphy [6]. However, this case shows ectopic gastric mucosa in the rare “ectopic Meckel’s diverticulum” in the upper mid abdomen, which was surgically proven to be at the mid ileum.
